# Pooled Analysis of the Accuracy of Xpert Ebola Assay for Diagnosing Ebola Virus Infection

**DOI:** 10.1155/2021/5527505

**Published:** 2021-05-17

**Authors:** Zhi-Yong Pan, Yan-Jun Wu, Ye-Xian Zeng, Hao Lin, Tian-Ao Xie, Ya-Ping Li, Ye-Ling Liu, Zhi-Jian He, Xu-Guang Guo

**Affiliations:** ^1^Department of Clinical Laboratory Medicine, The Third Affiliated Hospital of Guangzhou Medical University, Guangzhou 510150, China; ^2^Department of Clinical Medicine, The Third Clinical School of Guangzhou Medical University, Guangzhou 511436, China; ^3^Department of Clinical Medicine, The Second Clinical School of Guangzhou Medical University, Guangzhou 511436, China; ^4^Department of Clinical Medicine, The School of Mental Health of Guangzhou Medical University, Guangzhou 511436, China; ^5^Key Laboratory for Major Obstetric Diseases of Guangdong Province, The Third Affiliated Hospital of Guangzhou Medical University, Guangzhou 510150, China; ^6^Key Laboratory of Reproduction and Genetics of Guangdong Higher Education Institutes, The Third Affiliated Hospital of Guangzhou Medical University, Guangzhou 510150, China

## Abstract

**Background:**

West Africa has witnessed the unprecedented outbreak of Ebola virus disease (EVD). The Ebola virus (EBOV) can cause Ebola hemorrhagic fever, which is documented as the most deadly viral hemorrhagic fever in the world. RT-PCR had been suggested to be employed in the detection of Ebola virus; however, this method has high requirements for laboratory equipment and takes a long time to determine Ebola infection. Although Xpert Ebola is a fast and simple instrument for the detection of Ebola virus, its effect is still unclear. This study is aimed at evaluating the accuracy of Xpert Ebola in diagnosing Ebola virus infection.

**Methods:**

Using the keywords “Xpert” and “Ebola virus”, relevant studies were retrieved from the database of PubMed, Embase, Web of Science, and Cochrane. RT-PCR was employed as a reference standard to evaluate whether the study is eligible to be included in the meta-analysis. Data from these included studies were extracted by two independent assessors and were then analyzed by the Meta-DiSc 1.4 software to produce the heterogeneity of sensitivity (SEN), specificity (SP), positive likelihood ratio (PLR), negative likelihood ratio (NLR), and diagnostic advantage ratio (DOR) of the study. The results of pooled analysis were plotted, together with the summary receiver operating characteristic (SROC) curve plotted by calculating the area under the curve (AUC). Generated pooled summary estimates (95% CIs) were calculated for the evaluation of the overall accuracy of this study.

**Results:**

Five fourfold tables were made from the four studies that were included in the meta-analysis. The pooled sensitivity of Xpert Ebola was 0.98 (95% confidence interval (CI) (0.95, 0.99)), and the pooled specificity was 0.98 (95% CI (0.97, 0.99)). The pooled values of positive likelihood ratio was 53.91 (95% CI (12.82, 226.79)), with negative likelihood ratio being 0.04 (95% CI (0.02, 0.08)) and diagnostic odds ratio being 2649.45 (95% CI (629.61, 11149.02)). The AUC was 0.9961.

**Conclusions:**

Compared with RT-PCR, Xpert Ebola has high sensitivity and specificity. Therefore, it is a valued alternative method for the clinical diagnosis of Ebola virus infection. However, the Xpert Ebola test is a qualitative test that does not provide quantitative testing of EBOV concentration. Whether it can completely replace other methods or not calls for further evidences.

## 1. Introduction

Ebola virus pertains to the family Filoviridae [1, 2]. In 1976, its emergence in Africa induced a novel viral hemorrhagic fever, i.e., Ebola hemorrhagic fever. The Ebola virus, condemned as highly contagious, is transmitted between humans through contact with infected body fluids [3–9]. Severe Ebola virus infection reflects a systemic, destructive viral infection of multiple organs and multiple cell types [6]. Ebola virus disease (EVD) has posed challenges for its prevention and treatment and has seen countless attempts to understand its ecology and epidemiology, pathophysiology, and pathogenesis [6].

Technologies based on reverse transcription-polymerase chain reaction (RT-PCR) perform highly sensitive and specific detection of RNA viruses and have been suggested to be employed in the detection of Ebola virus [10, 11]. However, this method imposes strict requirements on each analytical stage and is restricted to labs. Furthermore, it takes a time ranging from 6 hours to 3 days to determine Ebola infection, resulting in a delay in treatment [12]. By contrast, the Xpert Ebola test detects Ebola virus by obtaining peripheral blood and saliva from oral swabs and venipuncture [13]. A specific kit (Xpert Ebola) for the EBOV Zaire strain targets highly conserved sequences in the nucleocapsid protein and glycoprotein genes [14]. Once the inactivated sample is added to the dedicated box and loaded onto the platform, no further actions are required from the operator [15]. Compared to PCR-based methods in the laboratory, the new Xpert Ebola technology is gaining momentum and attracting major attention. Whereas the Xpert Ebola detection method has not been introduced into China, there is considerable foreign literature documenting the contribution of Xpert Ebola combined with RT-PCR to the diagnosis of Ebola. Therefore, a meta-analysis was performed to evaluate the diagnostic efficacy of Xpert Ebola for Ebola virus, which may facilitate the development of the early diagnosis of Ebola virus.

## 2. Materials and Methods

### 2.1. Electronic Searches

All studies were published in Embase, PubMed, Web of Science (WOS), and Cochrane before October 25^th^, 2019, the databases of which were searched with the keywords “Ebola virus” and “Xpert”, and related researches were included. There were no language restrictions applied to this program.

### 2.2. Study Selection and Screening

Two investigators screened the full text of the potentially relevant publications independently, with the results cross-checked by the two investigators. Any discrepancy that appeared would be discussed to determine whether the publication be included or not. Should there be any inconsistency, a third investigator would be designated to assess the results. All qualified studies were involved in this analysis.

### 2.3. Inclusion and Exclusion Criteria

#### 2.3.1. Inclusion Criteria

The inclusion criteria are as follows: (1) the analytical studies of human samples; (2) studies that compared Xpert Ebola with RT-PCR in terms of the diagnostic accuracy of Ebola virus; the latter was employed as the gold standard; (3) studies from which sufficient data were extracted to construct 2 × 2 tables for calculating the sensitivity, specificity, and likelihood ratios; (4) studies with no less than 40 samples.

#### 2.3.2. Exclusion Criteria

The exclusion criteria are as follows: (1) duplicate studies; (2) abstracts, letters, conference abstracts, comments, letters, case reports, and reviews; (3) animal studies; (4) studies lacking for complete raw data, whose raw data not enough to construct 2 × 2 tables, or whose authors are unable to be contacted to obtain raw data.

### 2.4. Literature Screening

All the studies were screened and retrieved independently by two researchers, who checked the results after the screening process and determined the inclusion or exclusion of studies with inconsistent results after due discussion. If no agreement was arrived after the discussion, a third researcher would be assigned to evaluate the inconsistency; all the results were pooled together.

### 2.5. Data Extraction

The following data, namely, the title of the article, author, gold standard, country, year of publication, detection method, the number of included specimens, true positive, false positive, true negative, and false negative, were extracted and included in this study, with which the 2 × 2 tables were constructed.

### 2.6. Quality Assessment

Unified quality evaluation forms were made separately by the two researchers to evaluate the articles included, adopting the quality assessment of diagnostic accuracy studies (QUADAS-2) as the instrument [16]. Integrated with Review Manager 5.2 software, the QUADAS-2 tool consists of four parts, namely, reference standard, patient selection, flow and timing, and index test. The four parts were all assessed for the risk of bias, and the first three were also evaluated for the concerns regarding clinical applicability.

Any disagreement arising from this process was subject to communication with a third researcher.

### 2.7. Data Analysis

We followed the methods of Chen et al. [17]. Data was processed and analyzed with the Meta-DiSc software v.1.4 [18] to obtain the diagnostic odds ratio, negative likelihood ratio, sensitivity, positive likelihood ratio, specificity, and its corresponding 95% confidence interval (CI). Besides, a layered summary receiver operation characteristic (SROC) curve was constructed. The accuracy of the diagnosis of Ebola virus by Xpert Ebola was analyzed by the stochastic effect model and presented in the form of a forest map.

## 3. Results

### 3.1. Literature Filtering and Inclusion Process

A total of 28 studies (10 from WOS, 7 from PubMed, 11 from Embase, and 0 from Cochrane) were screened, and 15 duplicates were excluded. After browsing the titles and abstracts, according to the inclusion/exclusion, 4 articles were excluded (1 review, 2 conference abstracts, and 1 case report). By browsing the full text, 5 articles were further excluded in that 2 studies lacked the reference standard of RT-PCR, while for another 3, the original data was not sufficient enough to form complete 2 × 2 tables. Zero gray literature was detected during the second screening and the full-text browsing. Therefore, 4 studies that fully met the inclusion criteria were finally included (Figure S1).

### 3.2. Characteristics and Quality Assessment of Included Studies

These 4 articles covered the research results from 2015 to 2016 [14, 15, 19, 20], involving a total of 982 samples. Among the 4 articles, samples of 3 were only from semen, and 1 from both whole blood (WB) and semen. The characteristics of the study are summarized in Table 1.

**Figure 1:**
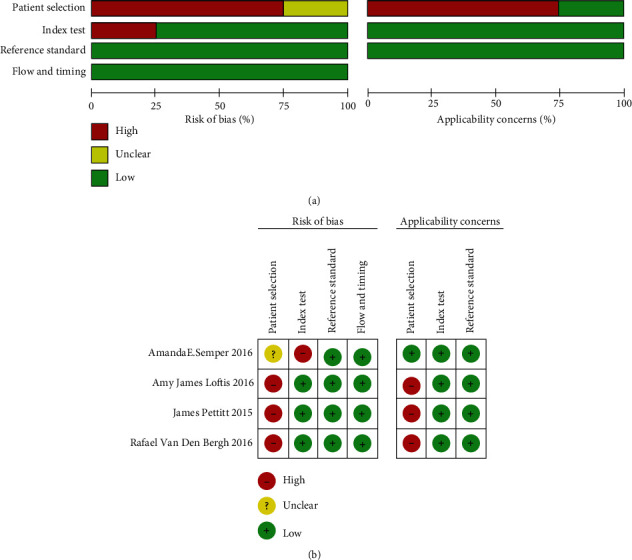
(a) Overall quality assessment of the included studies. (b) Quality assessment of the individual studies.

Methodological quality: the conclusion was arrived that most studies reveal a low risk of bias and a low concern regarding the applicability of the results. Four studies met the standard in all respects, and results of the quality assessment of individual studies are shown in Table 2, and the overall risk of bias and concerns of applicability of the four articles selected can be seen in Figure 1. In terms of patient selection, 3 studies (75%) were classified as a high risk for bias since their samples of patients were not enrolled consecutively and the unfavorable case-control design. In the respect of the index test, 1 study was believed to have a risk of bias because the index test results were interpreted with the reference standard. The remaining studies were not judged to be low risk. As for the assessment of reference standard, all of our studies displayed a low risk of bias since the reference standard results were interpreted without knowledge of the results of the index test according to data and quality evaluations. In the meantime, there was little concern regarding the applicability of all studies. In terms of timing and flow, all studies were considered to have a lower risk of bias because all patients were included in the analysis and all patients received the same reference standard, and there was an appropriate interval between the index test and the reference standard.

### 3.3. Threshold Effect Analysis

The Spearman correlation coefficient was 0.300 (<0.6), and *P* value was 0.624 (>0.05), which indicated that there was no threshold effect in our included studies.

### 3.4. Heterogeneity Analysis of Nonthreshold Effects

A forest plot was used to draw the ratios in a random pattern. As can be seen from Figure S2, the following values are obtained: Cochran *Q* = 2.41, *P* = 0.6605 (*P* > 0.05), inconsistency = 0.0% (inconsistency < 50%), showing no heterogeneity in nonthreshold effects.

### 3.5. SROC Curve

**Figure 2:**
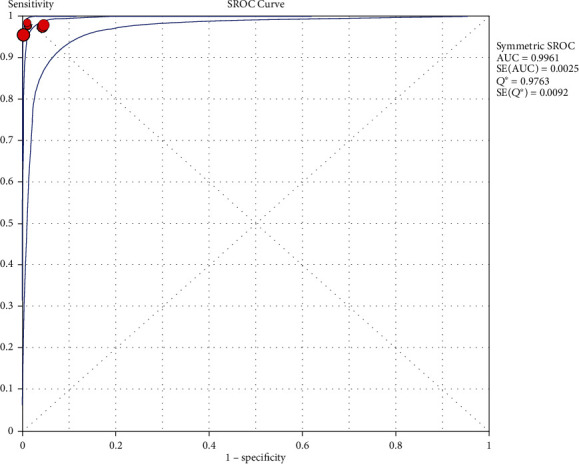
SROC curves of EVD detected by Xpert Ebola.

A random-effects model was adopted to fit the SROC curve. As shown in Figure 2, AUC = 0.9961 and the *Q* index is 0.9763 (SE = 0.0092). The *Q* index is the coordinate on the SROC curve with sensitivity = specificity and closest to the upper left corner. Combining data with Xpert Ebola displays a higher sensitivity for detecting Ebola. Therefore, it is believed that the detection of Ebola with Xpert Ebola has a higher accuracy.

### 3.6. Combine Analytic Results

**Figure 3:**
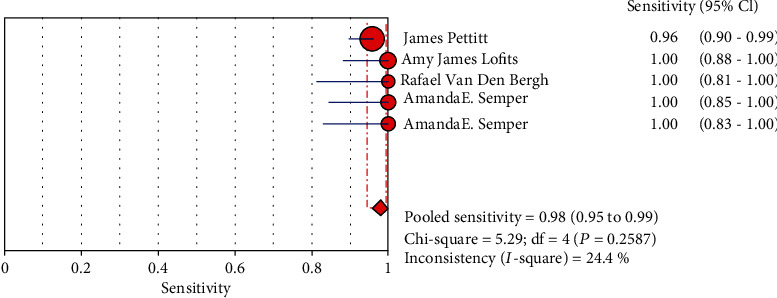
Forest plots for the pooled sensitivity of Xpert Ebola.

**Figure 4:**
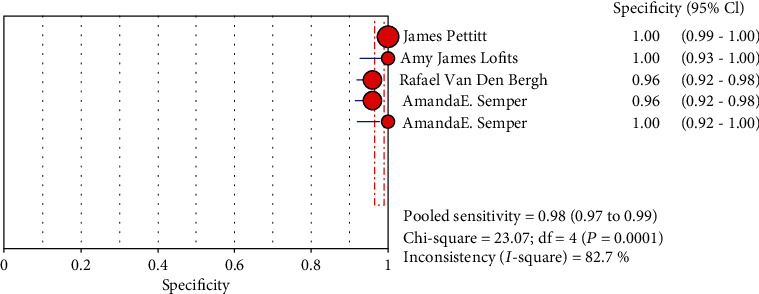
Forest plots for the pooled specificity of Xpert Ebola.

The results are shown in Figures 3, 4, S3, and S4. We detect our combined specificity, sensitivity, positive LR, and negative LR of Ebola virus. The values were 0.98 (95% CI (0.95, 0.99)), 0.98 (95% CI (0.97, 0.99)), 53.91 (95% CI (12.82, 226.79)), and 0.04 (95% CI (0.02, 0.08)); the combined diagnostic odds ratio was 2649.45 (95% CI (629.61, 11149.02)) by Xpert Ebola technology.

## 4. Discussion

Xpert Ebola was employed to statistically analyze the effect of Ebola detection, and it was compared with the gold standard in order to study the value of Xpert Ebola detection technology. In this research, a strict search was conducted with rigorous screening criteria, and finally, 4 articles were included. Quality evaluation results showed that the sensitivity and specificity of Xpert Ebola in the diagnosis of Ebola virus are both 0.98. The positive likelihood ratio was 53.91, while the negative likelihood ratio was 0.04, and the diagnostic ratio was 2649.45. Judging from the SROC AUC of 0.9961, Xpert Ebola is highly specific and sensitive for the diagnosis of Ebola virus. The SROC curve is located approximately at the upper left corner of the chart, which could be regarded as a larger space under the curve. Hence, Xpert Ebola is defined as highly accurate in the diagnosis of Ebola. However, heterogeneity in specificity is 82.7%. In the research of Pettitt's team [20], the heterogeneity mentioned therein might come from accidental unavoidable factors or caused by the limitations of experimental operations and consequences. For example, the mismatch of primers and probes may cause some Xpert Ebola positive samples to be negative by RT-PCR. This is caused by the genetic drift of EBOV's GP and NP genes. In the research of Pettitt's team [20] and Van den Bergh et al. [14], false positive samples have been shown to have relatively low viral loads, and these samples cannot be detected by conventional PCR methods, while by contrast, Xpert Ebola analysis can detect positive samples with low viral load. Therefore, Xpert Ebola diagnostic technology has the potential to detect early EVD.

At present, RT-PCR is widely adopted as the gold standard [14, 15, 20]. However, RT-PCR is highly demanding in terms of the equipment, operator, operating environment, and collection and storage of samples. The research of Pettitt's team [20] mentioned that traditional PCR analysis is to some extent exposed to the air, which increases the risk of introducing low-level pollutants that may interfere with RT-PCR results. Xpert Ebola, on the contrary, designed as an easy-to-use closed-box system avoids this concern. What is more, the sample analysis buffer of Xpert Ebola can inactivate Ebola virus and greatly reduce the risk of infection during the experiment. This also explains why traditional PCR analysis cannot be widely applied in large-scale primary hospitals. Another highlight of the Xpert Ebola detection method is that, as mentioned by Pettitt's team [20], it employs an internal algorithm to report RNA detection, which eliminates the possibility of bias introduced by operators. In a word, Xpert Ebola presents high sensitivity and specificity for detecting Ebola virus in whole blood and semen.

Loftis et al. [19] mentioned that they were unable to evaluate clinical samples at that time, and no further evaluation of biosafety procedures had been performed with Xpert Ebola's test, and that live EBOV had not been completely discontinued.

It was documented by James Pettitt's team [21] that most of the samples tested by Xpert Ebola are semen samples right now. However, lacking of experience with testing semen samples will lead to the possibility of heterogeneity in negative or positive EBOV tests when these samples are used to filter survivors and analyze these results.

Loftis et al. [19] mentioned that the Xpert Ebola test as a qualitative test is unable to provide quantitative detection of EBOV concentration; thus, it may have certain limitations in terms of the diagnosis, therapy, care, or determination of the infection and/or transmission of EVD. It is not appropriate to widely implement Xpert Ebola technology at this time since a precision pipette for sample inactivation processing is still required.

Our study also has certain limitations. First of all, we only extract data from English databases. Although we use a comprehensive retrieval strategy, we are not sure that we will not miss any articles. Second, the number of included articles was small and no publication bias analysis was conducted. Besides, in those literatures included in our research, mainly male subjects were tested, which may lead to inadequacy of the study to some extent.

## 5. Conclusion

To sum up, RT-PCR, adopted as the golden standard for detecting Ebola virus in whole blood in this research, has long been used for clinical diagnosis of Ebola infection. Our study, as a valuable reference, provides another method, i.e., Xpert Ebola, which performs effectively in the detection of Ebola in semen with high sensitivity and specificity as well as time-efficiency and convenience. However, at this stage, Xpert Ebola as a detection method of Ebola has not been widely documented. In those literatures included in our research, mainly male subjects were tested, which may lead to inadequacy of the study to some extent.

## Figures and Tables

**Table 1:** Characteristics of the included 4 studies.

Authors	Study design	Location	Source of strains	Reference standard	Ebola virus				Blinded review or interpretation	The characteristics of the controls
					TP	FP	FN	TN		
James Pettitt [19] (2015)	Prospective cohort study	America	402 semen	Ebola Zaire target 1 (EZ1) and major groove binder RT-PCR	94	0	4	308	Yes	Samples were collected from uninfected individuals
Amy James Loftis [20] (2016)	Prospective cohort study	America	80 semen	Traditional PCR	30	0	0	50	Yes	Samples were procured commercially from healthy donors
Rafael Van den Bergh [14] (2016)	Prospective cohort study	Belgium	218 semen	A routine in-house Ebola PCR	18	8	0	192	Yes	Suspected EVD patients for routine diagnosis between May 2 and July 4, 2015
Amanda E. Semper [15] (2016)	Prospective cohort study	England	218 WB	Reverse transcription PCR, Trombley	22	8	0	181	Yes	Residual diagnostic specimens remaining after clinical testing from suspected or confirmed EVD patients between April 1 and July 20, 2015
Amanda E. Semper [15] (2016)	Prospective cohort study	England	64 semen	Reverse transcription PCR, Trombley	20	0	0	44	Yes	Residual diagnostic samples remaining after clinical testing from suspected or confirmed EVD patients between March 7 and July 20, 2015, some of which had been frozen before use

TP: true positive; TN: true negative; FP: false positive; FN: false negative; WB: whole blood.

**Table 2:** QUADAS-2 results for each study included in the meta-analysis.

QUADAS-2												
Author	Year	1	2	3	4	5	6	7	8	9	10	11
James Pettitt [19]	2015	N	UC	Y	Y	UC	Y	Y	Y	Y	Y	Y
Amy James Loftis [20]	2016	Y	N	Y	Y	N	Y	Y	Y	Y	Y	Y
Rafael Van den Bergh [14]	2016	Y	N	UC	Y	N	Y	Y	UC	Y	Y	Y
AmandaE.Semper [15]	2016	Y	Y	UC	N	Y	Y	Y	Y	Y	Y	Y

Note: Y = yes; N = no; UC = unclear.

## Data Availability

All data generated or analyzed during this study are included in this published article and its supplementary information files.

## Abbreviations

EVD: Ebola virus disease
WB: Whole blood
AUC: Area under the curve
CI: Confidence intervals
DOR: Diagnostic odds ratio
NLR: Negative likelihood ratio
OR: Odds ratio
PCR: Polymerase chain reaction
PLR: Positive likelihood ratio
QUADAS: Quality assessment of diagnostic accuracy studies
SEN: Sensitivity
SP: Specificity
SROC: Summary receiver operating characteristic.

## Supplementary Materials

Supplementary Materials Additional file 1: Figure S1: flow diagram of selection of studies for the meta-analysis. Additional file 2: Figure S2: forest plots for the combined diagnostic OR of EVD. Additional file 3: Figure S3: forest plots for the pooled positive likelihood ratio of Xpert Ebola. Additional file 4: Figure S4: forest plots for the pooled negative likelihood ratio of Xpert Ebola.
