# Supplementary material for: Pooled Analysis of the Accuracy of Xpert Ebola Assay for Diagnosing Ebola Virus Infection
Source: Biomed Res Int. 2021 May 17;2021:5527505. doi: 10.1155/2021/5527505 (PMC8147515; doi:10.1155/2021/5527505)
Supplement: Supplementary Materials — Additional file 1: Figure S1: flow diagram of selection of studies for the meta-analysis. Additional file 2: Figure S2: forest plots for the combined diagnostic OR of EVD. Additional file 3: Figure S3: forest plots for the pooled positive likelihood ratio of Xpert Ebola. Additional file 4: Figure S4: forest plots for the pooled negative likelihood ratio of Xpert Ebola. [file 5527505.f1.doc]

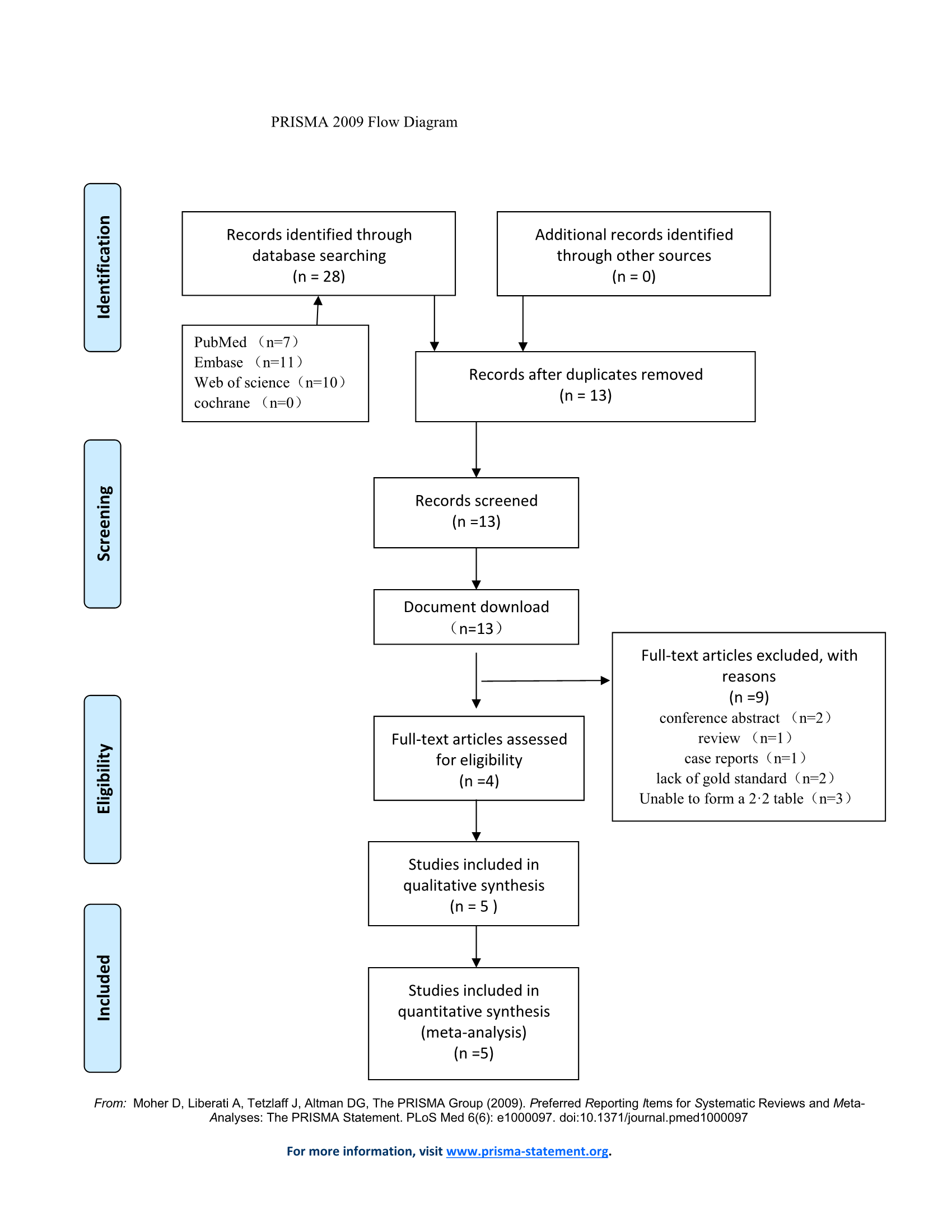


Figure S1: Flow diagram of selection of studies for the meta-analysis


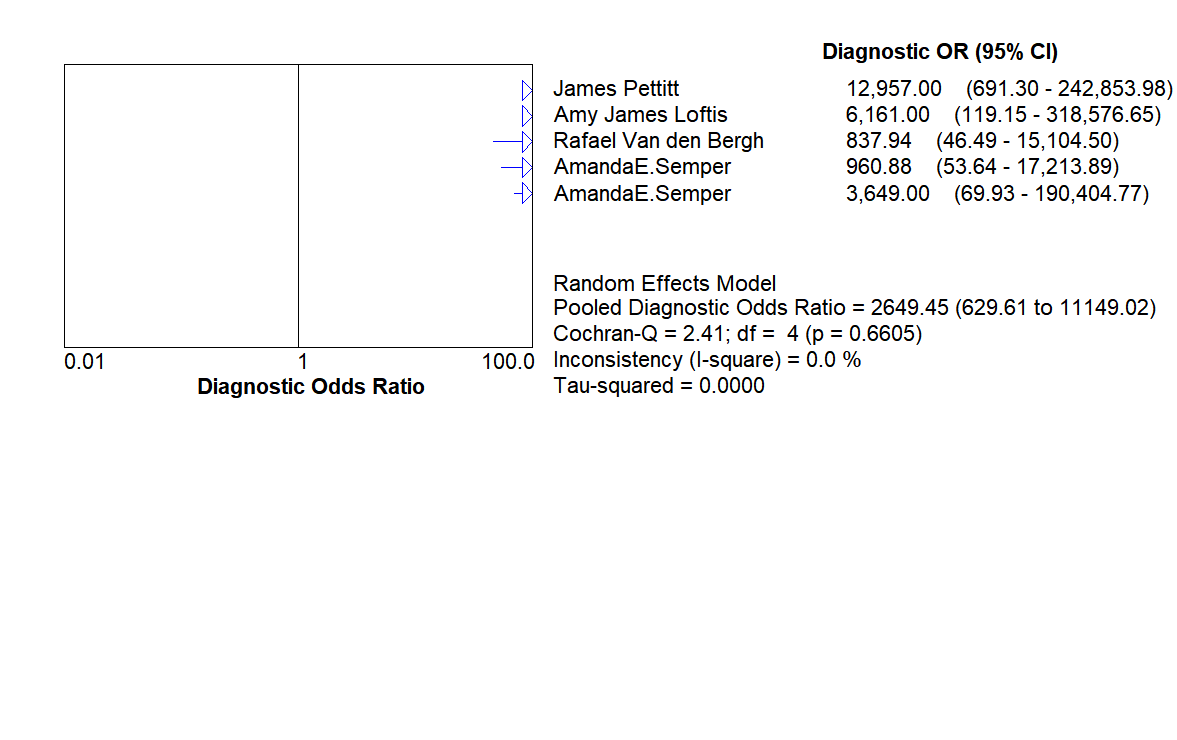


Figure S2: Forest plots for the combined diagnostic OR of EVD.


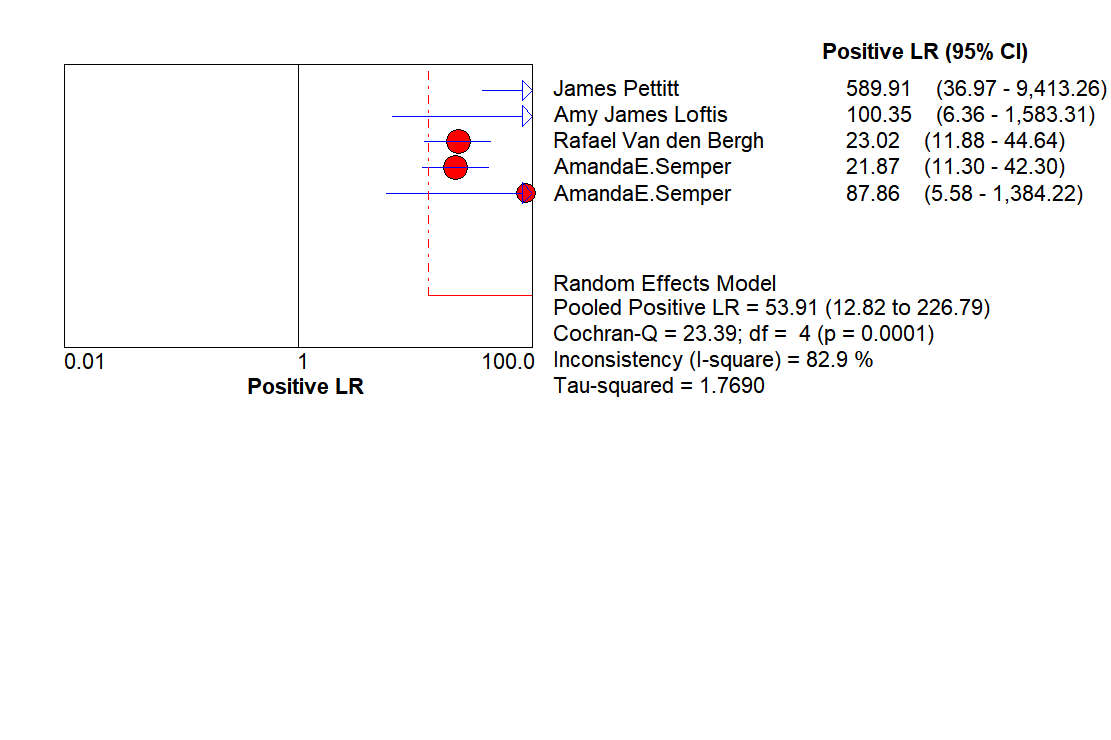


Figure S3: Forest plots for the pooled positive likelihood ratio of Xpert Ebola.


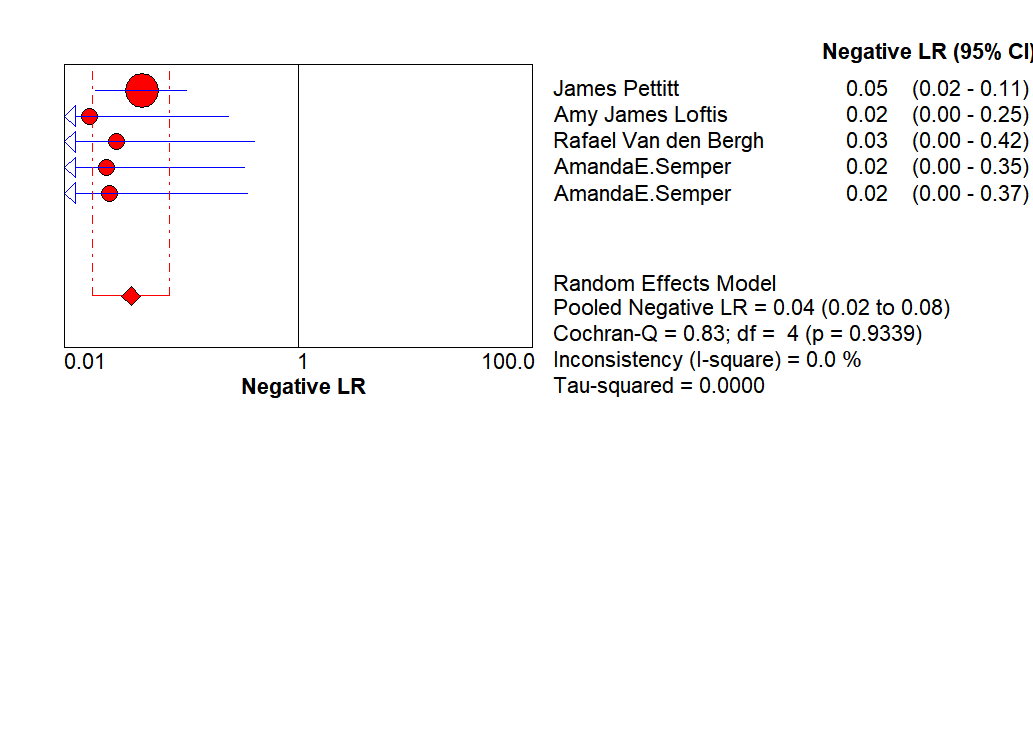


Figure S4: Forest plots for the pooled negative likelihood ratio of Xpert Ebola.
